# Self-Medication Practices and Associated Factors Among Health-Care Professionals in Selected Hospitals of Western Ethiopia

**DOI:** 10.2147/PPA.S244163

**Published:** 2020-02-20

**Authors:** Ginenus Fekadu, Dinka Dugassa, Getandale Zeleke Negera, Tilahun Bakala Woyessa, Ebisa Turi, Tadesse Tolossa, Getahun Fetensa, Lemessa Assefa, Motuma Getachew, Tesfaye Shibiru

**Affiliations:** 1 Department of Pharmacy, Institute of Health Sciences, Wollega University, Nekemte, Ethiopia; 2 School of Pharmacy, Institute of Health, Jimma University, Jimma, Ethiopia; 3 Department of Midwifery, Institute of Health Sciences, Wollega University, Nekemte, Ethiopia; 4 Department of Public Health, Institute of Health Sciences, Wollega University, Nekemte, Ethiopia; 5 Department of Nursing, Institute of Health Sciences, Wollega University, Nekemte, Ethiopia; 6 Department of Pediatrics and Child Health, Wollega University Referral Hospital, Nekemte, Ethiopia

**Keywords:** self-medication, self-medication practice, health care professionals, Ethiopia

## Abstract

**Background:**

Even though the type, extent and reasons for self-medication practice (SMP) vary, globally self-medication (SM) is rising to relieve burdens on health services. However, inappropriate SMP results in economic wastes, damage of vital organs, incorrect therapy selection, risk of adverse drug reactions and development of antimicrobial-resistant pathogens. These consequences have severe implications including legal, ethical and quality of health-care delivery. Temporal increment and high prevalence of SM among health professionals is also a major bottleneck for Ethiopia. Hence, the study aimed to assess the SM among health-care professionals (HCPs) in selected governmental hospitals of Western Ethiopia.

**Methods:**

An instiution-based cross-sectional study was conducted among 338 HCPs using a pre-tested and self-adminstered questionnaries from March 1 to 25, 2018. Simple random sampling was used to select study participants and SMP (yes or no) was the outcome of the study variable. Data were entered and analyzed using SPSS version 20. Crude and adjusted odds ratios (95% CI) were calculated and all results were deemed to be statistically significant when p < 0.05.

**Results:**

Among the 338 participants, 184 (54.4%) were females and the mean age of the study participants was 25±3.23 years. About 154 (45.6%) of them had work experience of less than 5 years and 49.7% were nurses by profession. The prevalence of SM was 73.4% with 3 months of recall for SM. Familiarity with medicines and ailments (46.8%) and mildness of illness (40.7%) were the most common reasons to self-medicate. The most frequently reported ailments were headache (37.1%) and gastric pain (29.8%). Analgesics (44.4%) and antibiotics (42.7%) were the most commonly used self-medicated categories of drugs. Female sex (Adjusted odds ratio [AOR] =2.13, 95% CI: 1.43–8.66), age 20–29 years (AOR=4.53, 95% CI: 1.01–14.45) and work experience of <5 years (AOR= 3.01, 95% CI: 1.32–11.71) were significantly associated with SMPs.

**Conclusion:**

The study revealed a high prevalence of SMP among HCPs. Sex, age, and work experience were significantly associated with SMPs. Hence, the use of prescription drugs without prescription should be discouraged and appropriate health education should be provided by all concerned bodies on the proper use of drugs.

## Background

The World Health Organization (WHO) defined self-medication (SM) as “the use of drugs to treat self-diagnosed diseases or symptoms, or the intermittent or continued use of a prescribed drug for chronic or recurrent disease or symptoms”.^1^–^3^ SM as one element of self-care involves the process of getting and consuming drugs without the advice of a physician either for diagnosis, treatment, prescription, surveillance or monitoring.^4^–^9^ It is also associated with the selection, procurement and use of medicines by individuals to treat self-diagnosed conditions as well as giving medications to other household members or dependent.^1^,^10^-^13^ Extensive complications resulting from SM among society are currently a matter of great importance.^14^,^15^

Even though the type, extent and reasons for self-medication practice (SMP) vary, globally SM is rising to relieve burdens on health services.^4^,^13^,^16^ Nowadays, the excessive use of SM is considered as one of the major health and socio-economic challenge in different countries.^14^ In developing countries, the easy availability of a wide range of drugs and inadequate health services increased the prevalence of SM.^3^,^17^ Although there may be a perception that SM is acceptable, it is unacceptable and illegal in most conditions.^18^

In recent years there has been an increasing trend in SM with non-prescription [over-the counter (OTC)] drugs available in pharmacies and retail outlets.^8^ As a result, medications may be approved as being safe for SM by the national drug regulatory authorities.^13^ Population-based analysis indicated that the prevalence of SMPs is alarmingly high among health-care professionals (HCPs), despite knowing the consequences and potential risks.^2^,^19^,^20^ The problem of SM among HCPs represents serious issues for both patients and physicians.^21^,^22^ The medical profession expects patients to seek appropriate medical help when they encounter significant problems with their health and yet HCPs do not behave in this way when it comes to their health.^23^,^24^ Additionally, when HCPs experience ill health, they disregard the advice they give their patients.^22^

SM is a major contributor to the health-care system and can be beneficial in various aspects if practiced appropriately. Convenience, economical, direct access to treatment, self-reliance in relieving minor symptoms, better use of clinical skills, easy access to medication and improving a person’s active role are some of the well-established advantages of SM.^25^–^29^ SM also recognized by WHO in certain conditions by reducing the burdens of already overloaded health-care systems, especially in developing countries.^26^ However, inappropriate SMP could have serious implications and global concern especially among elderly and special physiological conditions like pregnancy and lactation.^2^,^23^,^28^-^31^ It results in economic wastages, drug interactions, contraindication, prolong suffer, damaging of vital organs as well as responsible for the development of antimicrobial-resistant pathogens.^22^,^24^,^30^,^32^ It is also hazards to health because of the wrong diagnosis, inappropriate dosage regimen, risk of adverse drug reactions, incorrect choice of therapy, use of unnecessary expensive drugs, masking of severe disease as well as the risk of dependence and abuse.^11^,^16^,^27^-^29^,^31^,^32^ SM also increasing health-care costs and causing mortality and morbidity in different areas.^16^

The impact of the problem of SMPs among HCP is severe.^8^ This can lead to addiction and impaired functioning which is unprofessional and associated with unlawful behaviors that ultimately undermine the profession and pose a threat to the overall health and well-being of those involved.^22^ SM also increases the possibility of drug abuse and drug dependency as well as masks the signs and symptoms of underlying diseases, hence complicating the problem, and delaying diagnosis.^3^,^33^ These consequences of inappropriate SM among HCP have severe implications in legal, ethical, health defects as well as negative outcomes on patient and quality of health-care delivery.^8^,^22^ Apart from the adverse consequences, SM among HCPs may also lead to medical malpractice and negligence, lack of objectivity in diagnosis and treatment.^22^,^24^

Both general drug knowledge for the treatment of their disease and access to medications are potential factors for self-medication among HCPs.^19^,^20^,^34^ Other factors include the complaint of extensive demands on their time, issues of privacy and confidentiality.^20^ Additionally, multidrug prescribing, misuse of drugs, use of unnecessary expensive drugs, overuse of antibiotics and injections are common problems of irrational drug use by HCPs.^31^ Like any other person, HCP should also be encouraged through appropriate provision to enter the patient role. This is the only potential solution to decrease the high prevalence of SM among HCPs.^8^

Temporal increment and high prevalence of SM among HCPs is also a major bottleneck for Ethiopia.^2^,^10^ In Ethiopia, the extent of SMPs among HCPs is not yet fully known. The study aimed to assess the current situation of SMP among HCPs in governmental hospitals of Western Ethiopia.

## Methods and Participants

### Study Setting and Study Design

An institutional-based a cross-sectional study was conducted among HCPs in selected governmental hospitals of western Ethiopia from March 1 to 25, 2018. HCPs who were working in Nekemte Referal Hospital (NRH), Wollega University Referral Hospital (WURH), Sire General Hospital (SGH), and Arjo General Hospital (AGH) fulfilling inclusion criteria during the study period were included. NRH and WURH are located in Nekemte town 330 km away from Addis Ababa, capital city of Ethiopia.

### Eligibility Criteria

HCPs who were working in NRH, WURH, SGH and AGH who were willing to fill self-administered questionnaires and who were available during the data collection period were included in the study. However, HCPs who were not willing to fill the self-administered questionnaires were excluded.

### Sample Size and Sampling Technique

Sample size was calculated by using single proportion formula,^35^ which is n = z^2^ pq/d^2^, where z is estimated at 1.96 for a 95% confidence level, p was 67.5% of the estimated prevalence as per study by Sado et al in Nekemte, Ethiopia,^10^ and d is the level of acceptable error estimated at 5%. After considering 5% compensation for non-response, the final sample size was determined to be 354. Simple random sampling was used to select HCPs from selected governmental hospitals of Western Ethiopia.

### Data Collection Process

The self-administered semi-structured pretested data collection tool was adapted from previously published articles^8^,^10^,^31^,^36^ along with modifications to gather the necessary information on socio-demographic characteristics, prevalence, and determinants of SMP. The questionnaire was translated into Afan Oromo, the official language of the study zone by a panel of expert’s fluent in the language. To ensure the quality of data, the tool back-translated into English to check its consistency. The data were collected concerning the three-month recall of SMP during data collection period. Paper-based questionnaires were distributed to respondents and follow-up was made to retrieve the collected tool. SMP (yes or no) was the outcome of the study variable (dependent variable).

### Data Quality Control and Management

The clarity and completeness checkup of data collection tool was undertaken before the actual data collection. The translated data collection tool was pre-tested for its accuracy, completeness, and consistency before actual collection on 5% of HCPs who were working in Gimbi General Hospital. The data collection tool was commenced after a small amendment was made based on the results of the pretest. Furthermore, completeness, accuracy and clarity of the collected data were checked carefully. Collected data with incomplete information were excluded from the study to avoid the error.

### Data Processing and Analysis

Data were entered and analyzed using Statistical Package for the Social Sciences (SPSS) version 20. Descriptive data were presented in frequencies, percentages, means, and standard deviation with texts and tables. Variables found to be significant at the bivariate level (p<0.25) were considered for multivariate logistic regression to control the effect of potential confounders. Each variable was also entered back into the model one by one and removed again if it was not significant until the final model was achieved. The final model was checked to ensure that the model fitness is good. Crude and adjusted odds ratios (95% CI) were calculated and all results were deemed to be statistically significant when p < 0.05.

### Ethics Approval and Consent to Participate

This study was conducted in accordance with the Declaration of Helsinki. Ethical clearance was obtained from the Institute of health sciences of Wollega University with reference number IHSR/18/2018. A formal letter was written from the institute of health science, Wollega University to NRH, WURH, SGH and AGH for the permission to conduct the study. Those randomly selected study population were informed about the objectives and process of the study prior to data collection orally. The data gathered was anonymous and study participation was entirely voluntary. Written consent was obtained from each participant before the questionnaires were distributed and confidentiality was assured by avoiding identifiers from the data collection tool. Neither the case records nor the data extracted were used for any other purpose.

## Results

### Socio-Demographic Characteristics

A total of 354 health professionals were recruited with a response rate of 338 (95.5%). Among 338 study participants, 184 (54.4%) were females and the mean age of the study participants was 25±3.23 years. The modal age distribution was 20–29 year group accounted for 172 (50.9%) of the respondents. Approximately one-third (34.0%) of participants were between the ages of 30–39 years. About 154 (45.6%) have had work experience of less than 5 years and about half (49.7%) of HCPs were nurses by profession (Table 1).

**Table 1:** Socio-Demographic Characteristics of HCPs in Selected Governmental Hospitals of Western Ethiopia, March 1 to 25, 2018

Socio-Demographic Characteristics		Frequency (n=338)	Percentage (%)
Gender	Male	154	45.6%
	Female	184	54.4%
Marital status	Single	210	62.1%
	Married	106	31.4%
	Widowed	15	4.4%
	Divorced	7	2.1%
Age (years)	20–29	172	50.9%
	30–39	115	34.0%
	≥40	51	15.1%
Work experience (years)	<5	154	45.6%
	5–10	112	33.1%
	>10	72	21.3%
Professional type	Nurse	168	49.7%
	Pharmacist	48	14.2%
	Physicians	45	13.3%
	Midwives	36	10.7%
	Medical laboratory	27	8.0%
	Others*	14	4.1%

**Notes:** Anesthetics, X-ray technician, health officer, sanitarians, VCT counselors.

### Practices and Reasons for Self-Medication

About 248 (73.4%) study participants self-medicated for 3 months recall of SM. Familiarity with medicines and ailments 116 (46.8%) and mildness of illness 101 (40.7%) were the most common reasons to self-medicate among the study participants (Table 2).

**Table 2:** Reasons for Self-Medication by Participants Who Were Self-Medicated in Selected Governmental Hospitals of Western Ethiopia, March 1 to 25, 2018 (N=248)

Variables		Frequency (n)	Percentage (%)
Reason for self-medication	Familiarity with drugs (previous experience of treatment)	116	46.8%
	Mildness of illness	101	40.7%
	Lack of time (time saving)	77	31.0%
	Less cost (financial constraint)	51	20.6%
	Ease of accessibility	32	12.9%
	Privacy	21	8.5%
	Getting quick relief	14	5.6%
	Others*	9	3.6%

**Notes:** *Lack of interest in medical services, No primary physician nearby, Emergency use.

### Indications and Category of Drugs Used

The most common illness for SM was headache 92 (37.1%); followed by gastric pain (dyspepsia) 74 (29.8%) and cough and common cold 49 (19.8%). Analgesics 111 (44.4%) and antibiotics 106 (42.7%) were the most commonly used self-medicated categories of drugs (Table 3).

**Table 3:** Illness (Indications) and Medications for SM Reported by Study Participants Who Were Self-Medicated in Selected Governmental Hospitals of Western Ethiopia, March 1 to 25, 2018 (N=248)

Variables		Frequency (n)	Percentage (%)
Illness (indications) for self-medication	Headache	92	37.1
	Gastric pain (dyspepsia)	74	29.8
	Cough and common cold	49	19.8%
	Fever and chill	47	18.9%
	Allergy and asthma	28	11.3%
	Dysmenorrhea (painful menses)	19	7.6%
	Skin problems	11	4.8%
	Diarrhea	9	3.6%
	Urinary tract infection	6	4.4%
	Others*	11	3.6%
Categories of medicines used	Analgesics	111	44.4%
	Antibiotics	106	42.7%
	Anti-ulcer (antacids and proton pump inhibitors)	74	29.8%
	Topical applications (antifungal, anti-microbial, anti-histamines and analgesics)	38	15.3%
	Corticosteroids	24	9.7
	Oral contraceptive (OC)	19	7.6%
	Others**	9	3.6%

**Notes:** *Fungal or microbial infections, constipation, sore throat, eye disease, sore throat. **Anti-helminthes, laxatives, nasal/ear/eye drops.

### Factors Affecting Self-Medication Practice

Binary logistic regression was carried out, followed by multiple logistic regression to identify the independent predictors for SMP. On binary logistic regression analyses; sex, age, work experience, marital status and type of profession were associated with SMPs at p-value < 0.25. On multivariable logistic regression analysis; sex, age and work experience were significantly associated with SMPs at p < 0.05. The odds of practicing SM was about two times higher among females than males (AOR=2.13, 95% CI: 1.43–8.66, p=0.012). Those at the age group of 20–29 years were about 4.5 times more likely to practice SM than those who were ≥40 years of age (AOR=4.53, 95% CI: 1.01–14.45, p=0.003). Additionally, work experience was also associated with SMP in that those with a work experience of <5 years were three times more likely to practice SM than those with a work experience of >10 years (AOR= 3.01, 95% CI: 1.32–11.71, P=0.043) (Table 4).

**Table 4:** Factors Influencing SM Among HCPs in Selected Hospitals of Western Ethiopia, March 1 to 25, 2018

Variables		Self-Medication Practice		COR (95% CI)	AOR (95% CI)	p-value
		Yes (n=248)	No (n=90)			
Gender	Female	147	37	2.08 (1.41–6.54)*	2.13 (1.43–8.66)**	0.003
	Male	101	53	1		
Age (years)	20–29	144	28	3.90 (0.33–12.8)*	4.53 (1.01–14.45)**	0.012
	30–39	75	40	1.42 (0.19–6.47)		
	>40	29	22	1		
Marital status	Single	152	58	0.98 (0.64–1.53)*		
	Widowed/divorced	16	6	0.86 (0.41–1.26)		
	Married	80	26	1		
Work experience (years)	<5	116	38	2.58 (0.61–11.56)*	3.01 (1.32–11.71)**	0.043
	5–10	73	39	1.58(0.38–13.65)*		
	>10	39	33	1		
Profession	Nurse	117	51	1.72 (0.11–21.55)		
	Pharmacist	38	10	2.85 (1.08–9.78)*		
	Physicians	41	4	8.44 (0.81–21.64)*		
	Midwives	25	11	2.45 (1.33–4.01)		
	Medical laboratory	19	8	2.53 (0.60–8.53)		
	Others^#^	8	6	1		

**Notes:** ^#^Anesthetics, X-ray technician, health officer, sanitarians, VCT counselors. * Significantly associated at P-value< 0.25; ** significantly associated at P-value< 0.05.

**Abbreviations:** AOR, adjusted odds ratio; COR, crude odds ratio; CI, confidence interval.

## Discussion

This study investigated the prevalence of SM among HCPs and associated factors among selected hospitals of Western Ethiopia. Even though SM is a useful tool to treat minor ailments, improper SMP may lead to serious adverse drug reactions and possibly fatal consequences.^3^ Though not statistically significant, upon binary logistic regression physicians (COR: 8.44, 95% CI: 0.81–21.64) and Pharmacists (COR: 2.85, 95% CI: 1.08–9.78) were more practicing SM as compared to other professionals. This was similar with previous studies reporting SMP was more prevalent among physicians.^8^,^34^,^37^

According to this study, 73.4% of the study participants had practiced SM. Even if there were methodological differences, the result was relatively comparable with the study done in Malaysia 77.6%,^8^ Addis Ababa, Ethiopia 75.5%^27^ and Nekemte, Ethiopia 67.5%.^10^ But the prevalence was lower as compared to the study done in Pune 87.5%,^19^ Nigeria 81.8%^3^ and Eritrea 93.7%.^30^ Additionally, the prevalence was by far greater than those findings in Ethiopia reported from different cities and town’s including Sire 27.26%,^38^ Addis Ababa 26.6%,^39^ Mekelle University 42.67%,^13^ Gondar University 38.5%,^9^ Assendabo 39%,^40^ Nekemte (36.7%)^41^ and Jimma 27.6%.^42^ The types, extent, and reason for SM might vary from place to place due to study methodological differences and variation in socio-demographic characteristics of the study participants. The differences could also be as a result of the country’s drug laws or the electiveness of the drug regulating agencies of the countries where the studies were conducted. The studies of Boateng,^22^ Davidson and Schattner^43^, Gholab and Mohite^44^ also provide empirical pieces of evidence in support of this claim. Hence, SMP can be seen as generalized issue, both in developed and developing countries. Coordinated efforts are required to tackle the inappropriate and hazard consequences of SMP.

In our study site, the higher rate of SMP compared to other previous local studies in Ethiopia was due to the study participants where HCPs who had exposure to the drugs. This high rate of SMP among HCPs could be explained by poor or lack of drug control measures, regulatory policy, and planning. Across low and middle-income countries, poor access to health care, scarcity of trained HCPs, low standard of health care, easy accessibility of drugs, and patients’ misconception were some reasons for higher SMPs.^10^,^16^ Indeed, it has been detected that HCP especially physicians and pharmacists have great access to medication as well as their impressive knowledge of drug use as a basic pointer to the high prevalence rate of SMP.^8^

The study revealed that familiarity with medicines and ailments as well as the mildness of illness were major reasons for practicing SM. This finding was correlated with previous reports done in different areas.^2^,^8^,^9^,^13^,^19^,^22^,^27^ This means that HCPs particularly physicians had imposed their subjective judgment in determining both their diagnosis and treatment.^8^,^22^,^45^ Indeed, it has been observed that HCPs get access to medication as well as their knowledge of drug use is a major reason for the high rate of the SMPs. Balbisi and Ambiza also revealed that pharmacists and physicians are among the HCP with the greatest access to medications and having an impressive knowledge of prescription drugs and their use in the treatment of various drug therapies increases the potential of SM.^46^ Boateng et al also suggested that higher professional experience will be more likely to contribute to the knowledge of appropriate medication and familiarity of treatment options by the respondents and hence, increase the rate of SM.^22^ About 40.7% of the study participants reported that their illness condition was not serious enough to merit seeing a physician. The low severity of symptoms of illness was also frequently reported in the previous study by Ali et al.^8^ Other studies reported different reasons for SMP. For example, cost and convenience,^16^,^47^ cost and mildness of illness,^38^ ease of accessibility and saving time 208 (24.4%),^30^ and financial constraints and familiarity with medicines^10^ were the major reasons of SMPs. The difference for the reasons for SMP might be due to the difference in socio-demographic factors, study setup, study design and eligibility criteria involved.

The most common illness for self-medication was headache (37.1%), comparable findings were reported by previous studies.^8^,^9^,^13^,^38^ Headache is mostly mild to moderate diseases treated with OTC like analgesics, and in most of the cases, it does not require further diagnosis and treatment unless it is secondary to other diseases. Analgesics (44.4%) and antibiotics (42.7%) were the most commonly used categories of medicines correlated with previous study findings.^2^,^3^,^8^-^10^,^13^,^19^,^30^,^39^ Analgesics were the most common class of medications used in the SMPs because such drugs are used to treat simple common illnesses like headache, fever and pain. This was due to many analgesics are OTC drugs that are by law obtainable without prescriptions.

On the contrary, Andualem and Gebre-Mariam reported a high rate of antibiotics (84%) were used for SM.^7^ Antibiotics are susceptible to the risk of misuse and yet they are often exposed to the high rate of SMPs.^48^ This might imply that antibiotics could be obtained from drug retail outlets and pharmacies without prescription though they are prescription-only drugs.^8^,^13^ HCPs use antibiotics as OTC because of the poor practice of regulatory agencies and individuals acquiring antibiotics to sell on the black market as most of the HCPs feel that they will save money and time.^16^ This can lead to substantial adverse drug reactions, antibiotic resistance, treatment failure, and drug-related toxicity.^3^,^13^ Types and rates of SM drugs vary along with time and area due to differences in country’s approved OTC drugs, accessibility of the drugs, available regulations, disease and patient’s condition as well as experiences of the health-care providers.

On multivariable logistic regression analysis sex, age and year of service were significantly associated with SMPs. It was found that females were found to be more likely to practice SM than males. This was comparable to studies conducted in United Arab Emirates,^49^ Nagpur, India,^50^ Karad, India,^44^ Kolladiba, Ethiopia,^51^ and Arsi University, Ethiopia.^36^ The higher SMP among females might be related to the physiological difference they have and how they react to different diseases. For example, females have more disease burden due to physiological and biological factors including pains related to maternal and menstrual effects. However, this issue has to be explored with further research for possible explanatory reasons.

The study also revealed the relationship between work experiences with a rate of SM. Those participants with a work experience of <5 years were more likely to practice SM than those with a work experience of >10 years. This was comparable with previous studies that revealed less experienced health professionals had more tendency to practice SM.^8^,^49^ This might be due to HCP with less years of experience assume themselves they are sufficient and confident to treat themselves rather than seeking other HCPs. As time progresses with increased work experiences, HCP gained the knowledge of the medication use including ethical and related adverse events. Hence, HCPs with better work experience have better drug knowledge on the side-effects and consequence of improper SMP. Thus, experienced HCP focus on treatment confirmed by different kinetic investigations. However, there was no significant establishment between the years of professional experience and SMPs among respondents as per a study report by Ali et al in Malaysia.^8^ Professional experience could also be directly associated with drug knowledge and familiarity with treatment options. The difference might be due to differences in socio-demographic, eligibility criteria and study design utilized.

The study also indicated that the higher rate of SM was practiced by the participants at the lower age category. Those study participants at the age of 20–29 years were about 4.5 times more likely to practice SM than those who are at the age of >40 years. This was comparable with the previous study conducted by Esan in Nigeria where SM was found to be significantly associated with age (*p* =0.021).^3^ In the real sense, it was expected that the more practical work experience, the more likely to self-medicate. But this study was contrary to finding by Dorcas poku Boateng which identified that a higher rate of self-medication was practiced by higher respondent age group (46–60 years).^22^ The difference might be due to the study design, community culture and available health-care facility to treat patient’s cases. Other reasons for SMP within younger and less experienced HCPs might also be related to higher workload and time pressures, demanding families and personal lives (eg, continuous studying).

### Limitation of the Study

This study had some limitations. First, the study was conducted in a specific context, relatively small sample size and therefore more studies in different contexts with larger numbers and measuring observed behaviors would help to validate or refute the findings. The study was carried out among HCPs working in the public health facilities only. Hence, the study participants may not be representative of other HCP globally and generalization of the findings to other areas should be with caution. Additionally, 3 months recall period was used to collect information; hence, it might be subjected to recall biases. The analyses were also based on self-report with the possibility of over and under-reporting. Furthermore, the cross-sectional study design used did not allow for the causal–effect relationship to be inferred or concluded from the results of the study. Finally, the provision of socially acceptable responses by the study participants may lead to unique responses.

## Conclusion

The study revealed that the prevalence of SM was relatively higher among the study participants compared to previous local studies in Ethiopia. Analgesics and antibiotics were the most commonly used categories of drugs. Headache and gastric pain (dyspepsia) were the most common illnesses seeking for SM. Familiarity with medicines and ailments and mildness of illness were the most common reasons for SM. The professional exposure to drugs, knowledge of their illness and treatment choice remains as the fundamental contributor to SM practice among HCPs. Sex, age and work experience were significantly associated with SMPs. Although appropriate SMP is one of the components of self-care adopted by the WHO, irrational use is very likely to bring serious health consequences.

To overcome the spate of SMPs among HCPs, functional policy initiatives are required. Drug regulatory and health authorities have to dedicate some resources to raise awareness of the HCPs and the general public. Hence, continues and evidence based education should be provided on the advantages and disadavantages of SM for different stake-holders. Additionally, health education on medication through different media outlets and in health institutions has paramount importance to promote the appropriate use of drugs. Governmental health-care facilities must provide well-motivated, congenial and improved health-care services for HCPs. The implications of SMPs among HCPs and the consequences should also be introduced to future candidate HCPs like students of medical and health sciences. Finally, the use of prescription drugs without prescription should be discouraged and appropriate health education should be provided to all concerned bodies to raise the awareness of the HCPs on appropriate utilization of drugs and antimicrobials in particular. Thus, HCPs must be encouraged to enter the patient role when they are ill.

## Abbreviations

AGH, Arjo general hospital; HCP, health-care professional; NRH, Nekemte referral hospital; OTC, over the counter; SGH, Sire general hospital; SM, self-medication; SMP, self-medication practice; WHO, World health organization; WURH, Wollega university referral hospital.

## Data Sharing Statement

The datasets used and/or analyzed during the current study are available from the corresponding author on reasonable request.

## References

[1] World Health Organization. *Guidelines for the Regulatory Assessment of Medicinal Products for Use in Self-Medication*. Geneva: World Health Organization; 2000.

[2] Sisay M, Mengistu G, Edessa D. Epidemiology of self-medication in Ethiopia: a systematic review and meta-analysis of observational studies. *BMC Pharmacol Toxicol*. 2018;19(1):56. doi:10.1186/s40360-018-0248-8 30201045 PMC6131789

[3] Esan DT, Fasoro AA, Odesanya OE, Esan TO, Ojo EF, Faeji CO. Assessment of self-medication practices and its associated factors among undergraduates of a private University in Nigeria. *J Environ Public Health*. 2018;2018:1–7. doi:10.1155/2018/5439079 PMC6317103 30671097

[4] World Health Organization. *Role of Pharmacists in Self-Care and Self-Medication.the Fourth Consultative Group Meetings on the Role of the Pharmacist in the Health Care System Organized by WHO in Collaboration with the International Pharmaceutical Federation*. The Hague,The Netherlands: World Health Organization; 1998.

[5] Montastruc J, Bagheri H, Geraud T, Lapeyre-Mestre M. Pharmacovigilance of self-medication. *Therapie*. 1997;52(2):105–110. 9231503

[6] Hughes CM, McElnay JC, Fleming GF. Benefits and risks of self medication. *Drug Safety*. 2001;24(14):1027–1037. doi:10.2165/00002018-200124140-00002 11735659

[7] Andualem T, Gebre-Mariam T. Self-medication practices in Addis Ababa: a prospective study. *Ethiop J Health Sci*. 2004;14(1):1–10.

[8] Ali AN, Kai JTTK, Keat CC, Dhanaraj S. Self-medication practices among health care professionals in a Private University, Malaysia. *Int Curr Pharm J*. 2012;1(10):302–310. doi:10.3329/icpj.v1i10.11846

[9] Abay S, Amelo W. Assessment of Self-medication practices among medical, pharmacy, health science students in Gondar University, Ethiopia. *J Young Pharm*. 2010;2(3):306–310. doi:10.4103/0975-1483.66798 21042491 PMC2964771

[10] Sado E, Kassahun E, Bayisa G, Gebre M, Tadesse A, Mosisa B. Epidemiology of self-medication with modern medicines among health care professionals in Nekemte town, western Ethiopia. *BMC Res Notes*. 2017;10(1):533. doi:10.1186/s13104-017-2865-5 29084581 PMC5663131

[11] Ruiz ME. Risks of self-medication practices. *Curr Drug Saf*. 2010;5(4):315–323. 20615179 10.2174/157488610792245966

[12] Kiyingi K, Lauwo J. *Drugs in the Home: Danger and Waste*. 1993. 8185788

[13] Gutema GB, Gadisa DA, Kidanemariam ZA, et al. Self-medication practices among health sciences students: the case of Mekelle University. *J Appl Pharm Sci*. 2011;1(10):183.

[14] Mohseni M, Azami-Aghdash S, Sheyklo SG, et al. Prevalence and reasons of self-medication in pregnant women: a systematic review and meta-analysis. *Int J Community Based Nurs Midwifery*. 2018;6(4):272. 30465000 PMC6226611

[15] Bello FA, Morhason-Bello IO, Olayemi O, Adekunle AO. Patterns and predictors of self-medication amongst antenatal clients in Ibadan, Nigeria. *Niger Med J*. 2011;52(3):153. doi:10.4103/0300-1652.86124 22083501 PMC3213744

[16] Haque M, Rahman NAA, McKimm J, et al. Self-medication of antibiotics: investigating practice among university students at the Malaysian National Defence University. *Infect Drug Resist*. 2019;12:1333–1351. doi:10.2147/IDR.S203364 31190922 PMC6529675

[17] Shankar P, Partha P, Shenoy N. Self-medication and non-doctor prescription practices in Pokhara valley, Western Nepal: a questionnaire-based study. *BMC Fam Pract*. 2002;3(1):17. doi:10.1186/1471-2296-3-17 12236905 PMC130019

[18] University of Florida. The legality and ethics of self-prescribing. *Drugs Ther*. 2006;20:7.

[19] Sajith M, Suresh SM, Roy NT, Pawar D. Self-medication practices among health care professional students in a tertiary care hospital, Pune. *Open Public Health J*. 2017;10:1. doi:10.2174/1874944501710010063

[20] Rosen IM, Christie JD, Bellini LM, Asch DA. Health and health care among housestaff in four US internal medicine residency programs. *J Gen Int Med*. 2000;15(2):116–121. doi:10.1046/j.1525-1497.2000.11218.x PMC1495333 10672115

[21] Montgomery A, Bradley C, Rochfort A, Panagopoulou E. A review of self-medication in physicians and medical students. *Occup Med (Chic Ill)*. 2011;61(7):490–497. doi:10.1093/occmed/kqr098 21727179

[22] Boateng DP. *Self-Medication Among Doctors and Pharmacists at the Korle Bu Teaching Hospital*. Kwame Nkrumah University of Science and Technology; 2009.

[23] Murray MD, Callahan CM. Improving medication use for older adults: an integrated research agenda. *Ann Intern Med*. 2003;139(5_Part_2):425–429. doi:10.7326/0003-4819-139-5_Part_2-200309021-00009 12965970

[24] Medical Council. *Guide to Professional Conduct and Ethics for Registered Medical Practitioners*. 7th ed. New Delhi: Medical Council Dublin; 2009.

[25] World Self Medication Industry (WSMI). *The Story of Self-Care and Self-Medication: 40 Years of Progress, 1970–2010*. Ferney-Voltaire, France; 2010.

[26] Sherazi BA, Mahmood KT, Amin F, Zaka M, Riaz M, Javed A. Prevalence and measure of self medication: a review. *J Pharm Sci Res*. 2012;4(3):1774.

[27] Shafie M, Eyasu M, Muzeyin K, Worku Y, Martin-Aragon S. Prevalence and determinants of self-medication practice among selected households in Addis Ababa community. *PLoS One*. 2018;13(3):e0194122. doi:10.1371/journal.pone.0194122 29579074 PMC5868796

[28] Patil SB, Vardhamane S, Patil B, Santoshkumar J, Binjawadgi AS, Kanaki AR. Self-medication practice and perceptions among undergraduate medical students: a cross-sectional study. *J Clin Diagn Res*. 2014;8(12):HC20. doi:10.7860/JCDR/2014/6788.3956 25653969 PMC4316275

[29] Bennadi D. Self-medication: a current challenge. *J Basic Clin Pharm*. 2013;5(1):19. doi:10.4103/0976-0105.128253 24808684 PMC4012703

[30] Tesfamariam S, Anand IS, Kaleab G, et al. Self-medication with over the counter drugs, prevalence of risky practice and its associated factors in pharmacy outlets of Asmara, Eritrea. *BMC Public Health*. 2019;19(1):159. doi:10.1186/s12889-019-6470-5 30727984 PMC6364400

[31] Solomon W, Abede G. Practice of self-medication in Jimma Town. *Ethiop J Health Dev*. 2003;17(2):111–116.

[32] World Health Organization. The benefits and risks of self-medication. *WHO Drug Inf*. 2000;14(1):1–2.

[33] McCabe SE, Teter CJ, Boyd CJ. Illicit use of prescription pain medication among college students. *Drug Alcohol Depend*. 2005;77(1):37–47. doi:10.1016/j.drugalcdep.2004.07.005 15607840

[34] Christie JD, Rosen IM, Bellini LM, et al. Prescription drug use and self-prescription among resident physicians. *JAMA*. 1998;280(14):1253–1255. doi:10.1001/jama.280.14.1253 9786376

[35] World Health Organization. Sampling methods and sample size In: *Health Research Methodology: A Guide for Training in Research Methods*. 2nd ed. Omi S, editor. World Health Organization, Regional Office for the Western Pacific Manila; 2001.

[36] Bekele SA, Argaw MD, Yalew AW. Magnitude and factors associated with self-medication practices among university students: the case of Arsi University, College of Health Science, Asella, Ethiopia: cross-sectional survey based study. *Open Access Lib J*. 2016;3(06):1.

[37] Allibone A, Oakes D, Shannon H. The health and health care of doctors. *JR Coll Gen Pract*. 1981;31(233):728–734. PMC1972241 7338866

[38] Jaleta A, Tesema S, Yimam B. Self-medication practice in sire town, West Ethiopia: a cross-sectional study. *Cukurova Med J*. 2016;41(3):447–452. doi:10.17826/cukmedj.237467

[39] Beza SW. Self-medication practice and associated factors among pregnant women in Addis Ababa, Ethiopia. *Trop Med Health*. 2018;46(1):10. doi:10.1186/s41182-018-0091-z 29743807 PMC5928590

[40] Suleman S, Ketsela A, Mekonnen Z. Assessment of self-medication practices in Assendabo town, Jimma zone, southwestern Ethiopia. *Res Social Adm Pharm*. 2009;5(1):76–81. doi:10.1016/j.sapharm.2008.04.002 19285292

[41] Sado E, Gedif T. Drug utilization at household level in Nekemte Town and surrounding rural areas, western Ethiopia: a cross-sectional study. *Open Access Lib J*. 2014;1(03):1.

[42] Worku S. Practice of self-medication in Jimma Town. *Ethiop J Health Dev*. 2003;17(2):111–116.

[43] Davidson SK, Schattner PL. Doctors’ health‐seeking behaviour: a questionnaire survey. *Med J Aust*. 2003;179(6):302–305. doi:10.5694/mja2.2003.179.issue-6 12964913

[44] Gholap MC, Mohite VR. Assess the self medication practices among staff nurses. *Indian J Sci Res*. 2013;4(1):81.

[45] Dabney DA, Hollinger RC. Illicit prescription drug use among pharmacists: evidence of a paradox of familiarity. *Work Occup*. 1999;26(1):77–106. doi:10.1177/0730888499026001005

[46] Balbisi EA, Ambizas EM. Self-prescribing of noncontrolled substances among pharmacists. *Am J Health Syst Pharm*. 2005;62(23):2508–2511. doi:10.2146/ajhp050007 16303907

[47] Nathan C, Cars O. Antibiotic resistance—problems, progress, and prospects. *N Engl J Med*. 2014;371(19):1761–1763. doi:10.1056/NEJMp1408040 25271470

[48] Richman PB, Garra G, Eskin B, Nashed AH, Cody R. Oral antibiotic use without consulting a physician: a survey of ED patients. *Am J Emerg Med*. 2001;19(1):57–60. doi:10.1053/ajem.2001.20035 11146021

[49] Sharif SI, Bugaighis LM, Sharif RS. Self-Medication practice among pharmacists in UAE. *Pharmacol Pharm*. 2015;6(09):428. doi:10.4236/pp.2015.69044

[50] Kasulkar AA, Gupta M. Self medication practices among medical students of a private institute. *Indian J Pharm Sci*. 2015;77(2):178. doi:10.4103/0250-474X.156569 26009650 PMC4442466

[51] Abrha S, Molla F, Melkam W. Self-medication practice: the case of Kolladiba town, north West Ethiopia. *Int J Pharma Sci Res*. 2014;5(10):670–676.

